# CREB Regulates Distinct Adaptive Transcriptional Programs in Astrocytes and Neurons

**DOI:** 10.1038/s41598-017-06231-x

**Published:** 2017-07-25

**Authors:** Luis Pardo, Luis Miguel Valor, Abel Eraso-Pichot, Angel Barco, Arantxa Golbano, Giles E. Hardingham, Roser Masgrau, Elena Galea

**Affiliations:** 1 Institut de Neurociències and Unitat de Bioquímica, Facultat de Medicina, Universitat Autònoma de Barcelona, Bellaterra, 08193 Barcelona Spain; 2 Instituto de Neurociencias, Universidad Miguel Hernández/Consejo Superior de Investigaciones Científicas, Sant Joan d’Alacant, 03550 Alicante Spain; 3 Unidad de Investigación, Hospital Universitario Puerta del Mar, Av. Ana de Viya 21, 11009 Cádiz, Spain; 4 UK Dementia Research Institute at The University of Edinburgh, Edinburgh Medical School, 47 Little France Crescent, Edinburgh, EH16 4TJ UK; 5 Deanery of Biomedical Sciences, Edinburgh Medical School, University of Edinburgh, Edinburgh, EH8 9XD UK; 6 ICREA, Pg. Lluís Companys 23, 08010 Barcelona, Spain

## Abstract

The cyclic AMP response element binding protein (CREB) is a primary hub of activity-driven genetic programs in neurons controlling plasticity, neurogenesis and survival. By contrast, the gene networks coordinated by CREB in astrocytes are unknown despite the fact that the astrocytic CREB is also activity-driven and neuroprotective. Herein we identified the transcriptional programs regulated by CREB in astrocytes as compared to neurons using, as study materials, transcriptome databases of astrocyte exposed to well-known activators of CREB-dependent transcription as well as publicly available transcriptomes of neuronal cultures. Functional CREB signatures were extracted from the transcriptomes using Gene Ontology, adult-brain gene lists generated by Translating Ribosome Affinity Purification (TRAP) and CREB-target gene repositories. We found minimal overlap between CREB signatures in astrocytes and neurons. In astrocytes, the top triad of functions regulated by CREB consists of ‘Gene expression’, ‘Mitochondria’, and ‘Signalling’, while in neurons it is ‘Neurotransmission’, ‘Signalling’ and ‘Gene expression’, the latter two being represented by different genes from those in astrocytes. The newly generated databases will provide a tool to explore novel means whereby CREB impinges on brain functions requiring adaptive, long-lasting changes by coordinating transcriptional cascades in astrocytes.

## Introduction

Since its discovery in the late 1980s, the cyclic AMP-response element-binding protein (CREB) is arguably the most widely studied transcription factor, its two best documented functions being the coordination of liver metabolism during cycles of feeding and fasting^1^, and the regulation of neuronal plasticity—defined as long-term adaptive changes—in several scenarios: development, memory acquisition and consolidation, addiction, circadian rhythms and natural regeneration after injury^2–5^.

Two lines of evidence support the notion that CREB regulates astrocytic functions as well. First, CREB-dependent transcription is activated in astrocytes by noradrenaline (NE), ATP, forskolin (FSK), tamoxifen and cinnamon^6–8^. NE and ATP act in a protein kinase C-dependent but calcium- and cyclic AMP-independent fashion, FSK activates adenylate cyclase and hence increases cyclic AMP content, whereas tamoxifen and cinnamon act *via* protein kinase A. Second, the targeted expression of a constitutively active CREB construct (VP16-CREB) in astrocytes is neuroprotective in focal acute brain injury^9^.

That is, both neuronal CREB and astrocytic CREB (hereafter neu-CREB and ast-CREB) are (i) activity-dependent (i.e., regulated by neurotransmitters), (ii) neuroprotective and (iii) activated by different signalling pathways. However, while the transcriptional programs regulated by neu-CREB have been well characterized *in vitro* and *in vivo* ^10–12^, there is little information about the gene targets of ast-CREB and the cellular origin of CREB-dependent transcriptional programs *in vivo*. Tools do exist to specifically activate CREB in neurons or astrocytes, but the related gene profiles have been characterized in whole brains without separating individual cells^11, 13^.

Herein we set out to identify the functional programs coordinated by CREB in astrocytes as compared to neurons. We generated transcriptome databases of rat cortex primary astrocyte cultures treated with NE or FSK in order to, respectively, activate cyclic AMP-independent and dependent pathways, or used over-expression of VP16-CREB, which promotes global expression of CREB-dependent genes regardless of upstream kinases^14^, and should thus faithfully unravel the complete population of ast-CREB-target genes. Since CREB-dependent transcription appears to be highly dependent on the stimulation context^15, 16^, we reasoned that the comparison of different transcriptomes, together with a stringent data mining using publicly available databases of adult genes, would lead us to the *core* functional signatures of ast-CREB as compared to neu-CREB. We found that ast-CREB and neu-CREB core signatures are utterly different, supporting a functional division of CREB in the brain between astrocytes and neurons.

## Results

### Analysis outline

**Figure 1:**
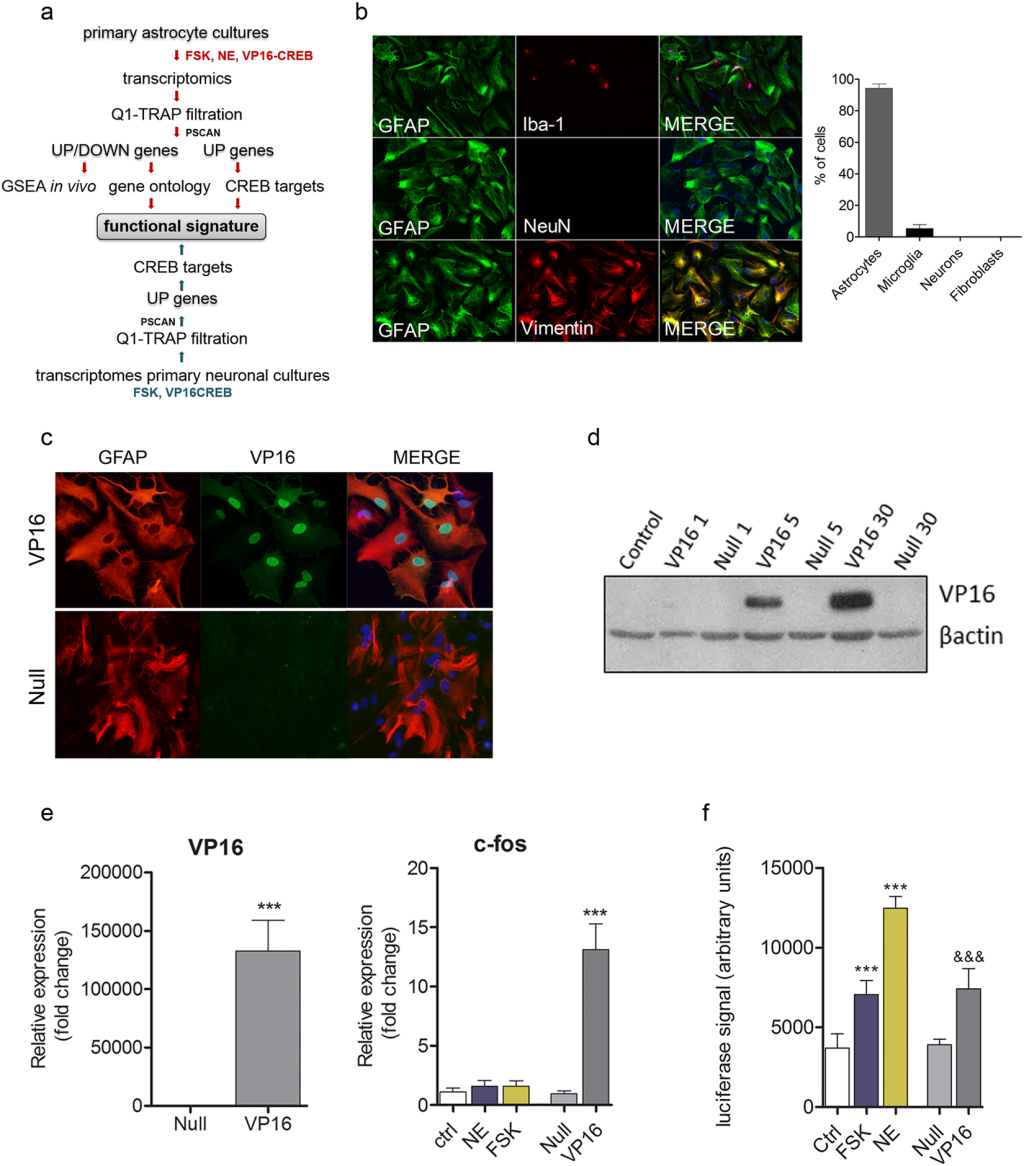
Outline and characterization of VP16-CREB over-expression in astrocytes. (**a**) Procedure outline. (**b**) Characterization of cellular composition. Cellular markers were GFAP (astrocytes), Iba1 (microglia), NeuN (neurons) and Vimentin, which labels both astrocytes (GFAP positive cells) and fibroblasts (GFAP negative cells). With respect to total DAPI counts, 95% of the cells were astrocytes, 5% microglia and no neurons or fibroblasts were detected−all Vimentin positive cells were GFAP positive as well. (**c**–**f**) Confirmation of VP16-CREB transduction. Astrocytes were incubated with Ad2/5-CMV-VP16-CREB or Ad2/5-CMV (Null) at MOIs within 1–30. (**c**) Immunodetection. The VP16-CREB is localized in the nucleus as expected for a CREB-like factor. Astrocytes and VP16-CREB were respectively identified by GFAP and VP16 immunostaining (viral infection at a MOI of 5). Infection with the Null virus showed no VP16-CREB expression. Images are representative of 3 independent experiments. (**d**) Western blots using anti-VP16 or anti-actin antibodies—the latter to control for equal protein load. VP16-CREB is 65 kd. Expression of VP16-CREB is MOI-dependent. The blot is representative of at least 3 independent experiments and is a cropped image. The full-length blot is presented in Supplementary Figure 1. (**e**) Left, robust up-regulation of VP16-CREB mRNA assessed by PCR in Ad2/5-CMV-VP16-CREB infected astrocytes but not in Null-infected ones. Data are means ± SEM of n = 3 independent determinations. (***) p < 0.001, Student’s T-test. Right, VP16-CREB induced *Fos* upregulation in Ad2/5-CMV-VP16-CREB infected astrocytes at a MOI of 5. *Fos* was not detected upon stimulation with NE and FSK because these stimuli are transient and *Fos* is a short-lived gene. Data are means ± SEM of 3 independent experiments; (***) p < 0.001, one-way ANOVA followed by Bonferroni. (**f**) Functional confirmation of VP16-CREB expression. VP16-CREB stimulates CRE-dependent transcription in luciferase-reporter assays (MOI = 5). FSK (1 μM) and NE (10 μM) were assayed in parallel as positive controls using 6-hour incubations. Data are means ± SD of an assay with four replicates representative of at least 5 independent experiments, (***^/&&&^) p < 0.001, one-way ANOVA followed by Bonferroni.

The study was carried out in the five stages depicted in Fig. 1a.

1. CREB-dependent transcription was activated in primary cortical rat astrocyte cultures with 10 μM NE, 1 μM FSK or VP16-CREB (multiplicity of infection or MOI of 5). Culture composition was 95% astrocytes (GFAP-positive cells) and 5% microglia (Iba1-positive cells) (Fig. 1b). Neurons (NeuN-positive cells) and fibroblasts (Vimentin positive/GFAP negative cells) were not detected.
2. Transcriptomic changes were characterized with Agilent DNA microarrays.
3. To minimize the impact of genetic signatures of neonatal stages and culture conditions on the resulting differentially expressed genes (DEG) (p < 0.05) we took two actions. First, we selected for the ensuing analyses DEGs more likely to be relevant *in vivo*. The lists were generated by filtration of DEG lists through the first quartile (Q1) of the translational ribosome affinity purification (TRAP), a repository of adult astrocyte genes that only contains genes undergoing effective translation^17^. Second, we examined whether DEG-Q1-TRAP lists were enriched in transcriptomes from adult brains with targeted expression of VP16-CREB in astrocytes^9^.
4. The DEG-Q1-TRAP lists for NE, FSK and VP16-CREB were processed independently to identify *functional* signatures by enrichment analysis using Gene Ontology (GO) with ClueGO v1.4 and ReviGO softwares.
5. Identification of *direct* ast-CREB gene target candidates and comparison with neu-CREB targets. Core signatures of ast-CREB-dependent gene candidates, defined as the set of genes common to all stimuli, were generated from the overlapping sections among DEG-Q1-TRAP NE/FSK/VP16-CREB, bioinformatic analysis of promoters^18^, and a database of genes containing CRE sites^19^. Likewise, the core of neu-CREB signature was obtained from common DEGs from FSK and VP16-CREB lists in neurons^10^ (ArrayExpress database; accession number E-MEXP-3167).

### VP16-CREB promotes CREB-dependent transcription in astrocytes

We have previously shown that NE and FSK induce CREB-dependent transcription in astrocytes^6^; here we confirmed that VP16-CREB does as well. Astrocytes infected with Ad2/5-CMV-VP16-CREB at MOIs of 5–30 efficiently transduced VP16-CREB, as deduced by the robust expression of VP16 mRNA and protein detected with Western blotting, immunocytochemistry and qPCR (Fig. 1c–e). That VP16-CREB activates CREB-dependent transcription was confirmed with luciferase assays, which showed a CRE-based activation comparable to that induced by FSK and NE (Fig. 1f), and by a ∼12-fold up-regulation of the *Fos* gene (Fig. 1e, right). Altogether, these analyses support the use of virally transduced VP16-CREB as a tool to induce CREB-dependent transcription in astrocytes. The viruses were used henceforth at a MOI of 5.

### Global transcriptome changes upon activation of ast-CREB-dependent transcription

The number of DEGs (p < 0.05) was 3279 genes in FSK-stimulated astrocytes and 2667 in the NE-treated group—relative to untreated controls—and 9884 in VP16-CREB over-expressing astrocytes with respect to Null conditions. We selected the genes present in the first quartile of the TRAP list of cortical astrocytes, ordered by expression level. The resulting genes will be referred to as Q1-TRAP genes and the discarded as REST (final lists in Supplementary data S2). The process reduced by approximately 3-times the number of genes per group, with the final numbers being: 3355 VP16-CREB, 1049 FSK, and 822 NE. Finally, we analyzed with PSCAN software the localization of direct CREB-targets throughout the transcriptomes. PSCAN scans gene promoters looking for motifs of transcription-factor binding sites—herein CRE sites—and statistically assesses which motifs are over-represented^18^.

**Figure 2:**
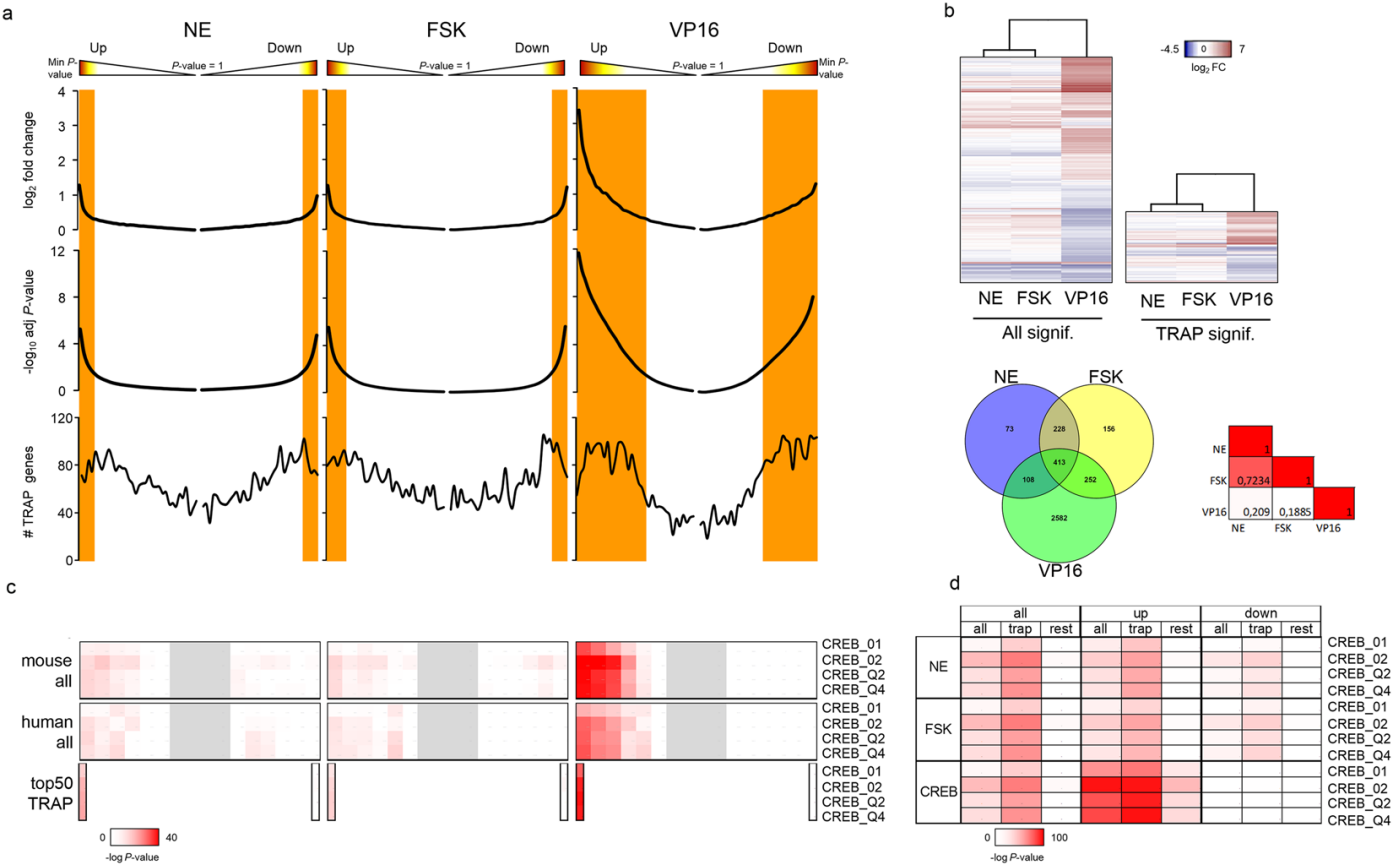
Transcriptome profiles. (**a**) Gene distribution according to fold change, adjusted p-values, and direction of change (UP or DOWN). Orange shades are regions of significance (adjusted *p*-value < 0.05). TRAP genes are genes in the first quartile (Q1) of the TRAP list. Bins of 250 genes were used to calculate the median of fold change and *p*-value, and to count the number of TRAP genes in each bin. (**b**) Heatmaps, Venn diagrams and Pearson correlations of differentially expressed genes. Top, gene clustering according to log_2_FC by Euclidean distance in the initial transcriptome (ALL genes) and in the list of genes filtered by Q1-TRAP list of adult astrocyte genes. Bottom, Venn diagrams of differentially expressed genes and Pearson correlations. There are major transcriptome changes upon VP16-CREB over-expression as compared to stimulation with FSK or NE, and the latter groups are highly similar. The same patterns are observed in ALL and TRAP genes. (**c**) PSCAN analysis of CRE-enrichment probability in ‘all’ and Q1-TRAP-filtered genes using promoters of mouse and human orthologs. The probability of CRE occurrence is highlighted in a spectrum of reds. Gray shades are transcriptome regions not analyzed. Rows are different matrices containing variations of CRE motifs. CRE-containing genes are enriched in the UP portion in all lists. (**d**) PSCAN analysis comparing ‘all’, ‘Q1-TRAP’ and ‘rest’, or remaining genes. CRE-containing genes are enriched in Q1-TRAP genes as compared to ‘rest’ genes.

Figure 2 shows the transcriptome profiles in the three groups before and after the Q1-TRAP filtration. VP16-CREB caused a dramatic change in the transcriptome as compared to NE and FSK, as shown by histograms of gene distribution, heatmaps and Venn diagrams (Fig. 2a,b). The proportion of up-regulated (UP) *vs* down-regulated (DOWN) genes was similar in the FSK and NE groups (47% *vs* 53%) and slightly deviated towards the UP section in the VP16-CREB group (64% *vs* 46%). That is, there was a slight predominance of UP genes after sustained activation, and the UP section reached larger fold-changes and had more significant p-values (Fig. 2a). Overall, the FSK and NE groups were very similar (Pearson’s correlation = 0.72), while the VP16-CREB group was markedly different from the other two (Pearson’s correlation = 0.18 *vs* FSK; 0.21 *vs* NE) (Fig. 2b, bottom right). These correlations were confirmed by the overlap of differentially expressed genes: 78% of the genes in the NE group were in the FSK group, and, conversely, 63% of the FSK genes were also in the NE group. The VP16-CREB group in turn included 79% of the genes present in the NE and FSK groups combined, whereas 61% of the genes were unique to VP16-CREB over-expression (Fig. 2b, Venn diagrams).

The PSCAN analysis revealed increased probability of CRE-site occurrence in the UP sections of all transcriptomes (Fig. 2c,d). The fact that highly similar results were obtained using mouse and human sequences supports the high conservation of CRE sites across species and demonstrates that no bias was introduced in using mouse genes. Of the three stimuli, VP16-CREB was the most efficient means to trigger the CREB-dependent transcriptional program, confirming that, in astrocytes as elsewhere, CREB is mostly a transcription activator.

The refinement of the global (ALL) list using the Q1-TRAP list did not introduce a bias in the transcriptome profiles since the distribution patterns of ALL genes were conserved in the Q1-TRAP gene group (Fig. 2a,b). FSK and NE groups were still highly similar (62% of the FSK genes were in the NE group and 77% of the genes in the NE group were in the FSK group); the VP16-CREB group included 95% of the genes in the NE and FSK groups; the distribution between UP and DOWN genes was approximately 50%, and CRE-sites were also enriched in the UP section of the Q1-TRAP list (Fig. 2c,d). Interestingly, CRE-sites were more enriched in the UP section of the Q1-TRAP list than in the UP section of the ALL list (Fig. 2c,d). This suggests that CREB-dependent genes are among the genes of highest expression in adult astrocytes.

### *In vivo* validation

**Figure 3:**
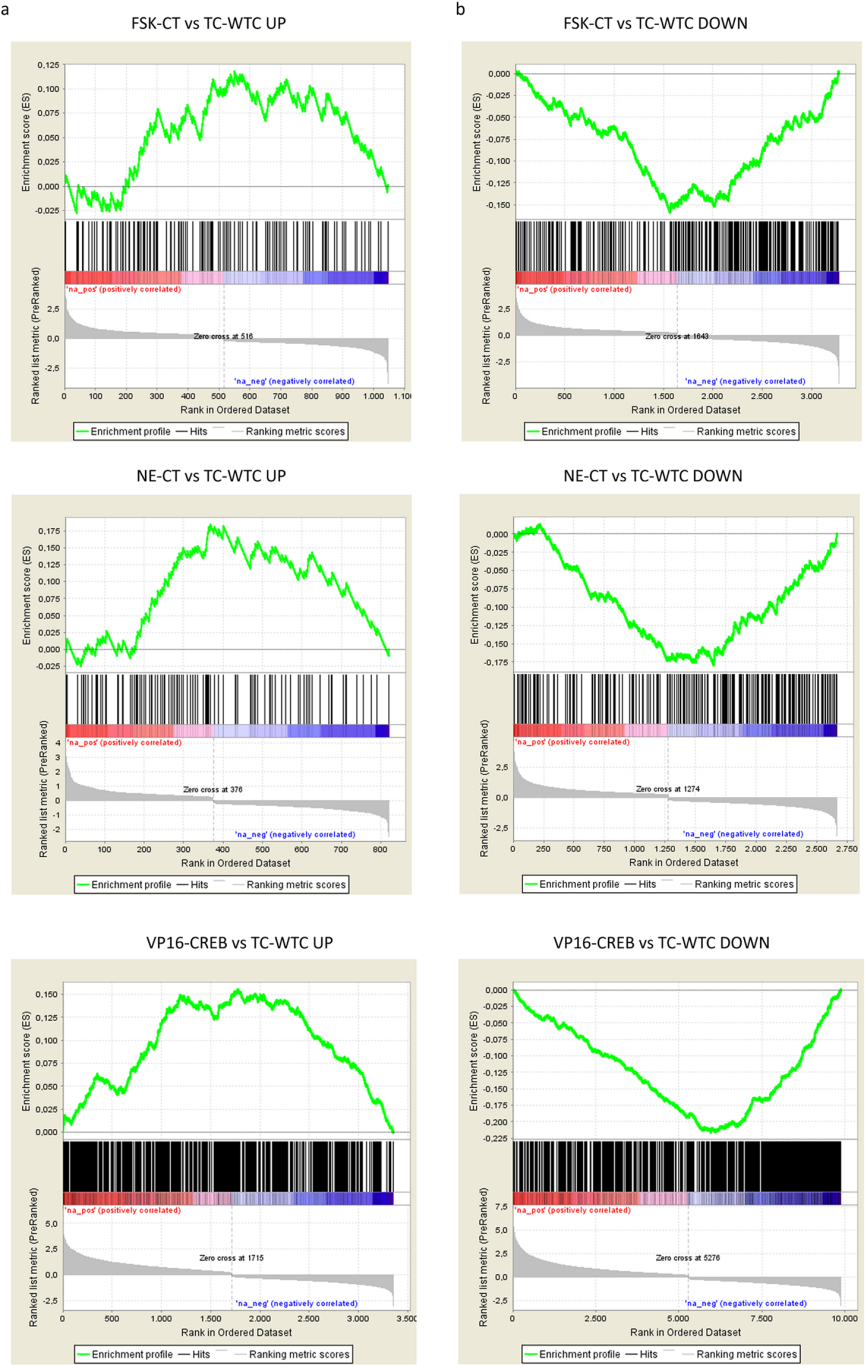
GSEA-based comparison of ast-CREB-dependent transcription *in vitro* and *in vivo*. Enrichment of DEG-Q1-TRAP databases in transcriptomes from mouse cortices with targeted expression of VP16-CREB in astrocytes (Gfa2-TtA/TetO-VP16-CREB mice). Transgen expression in mice was triggered by brain injury. GSEA comparisons were carried out between *in vitro* databases and the up-regulated (**a**) and down-regulated (**b**) fraction of transgenic *vs* wild-type mice (TC-WTC). The upper parts of the plots show enrichment scores, black lines represent the genes present in both lists (hits) and the bottom parts show the data sets ranked by gene expression (logFC).

In order to validate our strategy for extraction of adult genetic signatures by using Q1-TRAP, we compared DEG-Q1-TRAP lists with the transcriptomes of Gfa2-TtA/TetO-VP16-CREB transgenic mice, which express VP16-CREB in astrocytes linked to the activity of a *gfap*-based promoter, such that VP16-CREB expression is conditional to astrogliosis^9^. In the existing databases, VP16-CREB expression was achieved by acute injury caused by cryolesion (GSE68187). Using Gene Set Enrichment Analysis (GSEA)^20^, we compared the transcriptomes of transgenic *versus* wild-type mice subjected to cryolesion (TC-WTC group) with the *in vitro* gene lists (NE-CT, FSK-CT, VP16-Null) filtered by Q1-TRAP. GSEA showed a positive enrichment of *in vitro* gene lists in the portion of up-regulated genes in the group TC/WTC (‘positively correlated’ segment in Fig. 3a, statistics in Table 1), meaning that genes up-regulated upon NE/FSK treatment and VP16-CREB expression are also up-regulated in the cortex of transgenic mice upon targeted activation of CREB dependent transcription in astrocytes. The highest enrichment score (NES) was found—as expected—in VP16-CREB-Q1-TRAP (Table 1), while NE and FSK showed similar NES—although FSK enrichment was not statistically significant (Table 1). Conversely, the NES of *in vitro* genes in the down-regulated portion of TC-WTC was negative (‘negatively correlated’, Fig. 3b, Table 1), meaning that down-regulated genes, which plausibly represent cascades secondary to the primary wave of CREB-dependent transcription, are comparable *in vitro* and *in vivo*. The results support the use of *in vitro* tools to gain insight into direct and indirect transcriptional programs of CREB in astrocytes. For the sake of clarity, the group TC-WTC DOWN is equivalent to WTC-WT UP (Table 1) because the transgene reverts genotypical and phenotypical changes in lesioned wild-type mice back to normal^9^.

**Table 1:** GSEA of *in vitro* DEG-Q1-TRAP lists in Gfa2-TtA/TetO-VP16-CREB mice.

GENE SET	DATA SET	NES	NOM p-val
TC-WTC UP	FSK-CT	1.54	0.062
TC-WTC UP	NE-CT	2.10	0.004
TC-WTC UP	VP16-CREB	4.14	0.000
TC-WTC DOWN	FSK-CT	−3.12	0.000
TC-WTC DOWN	NE-CT	−3.15	0.000
TC-WTC DOWN	VP16-CREB	−7.07	0.000
WTC-WT UP	FSK-CT	−2.16	0.004
WTC-WT UP	NE-CT	−2.49	0.000
WTC-WT UP	VP16-CREB	−4.50	0.000

*In vitro* databases were compared with UP and DOWN genes in injured transgenic (TC) vs injured WT mice (WTC), and with UP genes in WTC vs WT mice. NES: normalized enrichment score. NOM p-val: normalized p-value.

### Functional signature of ast-CREB

**Figure 4:**
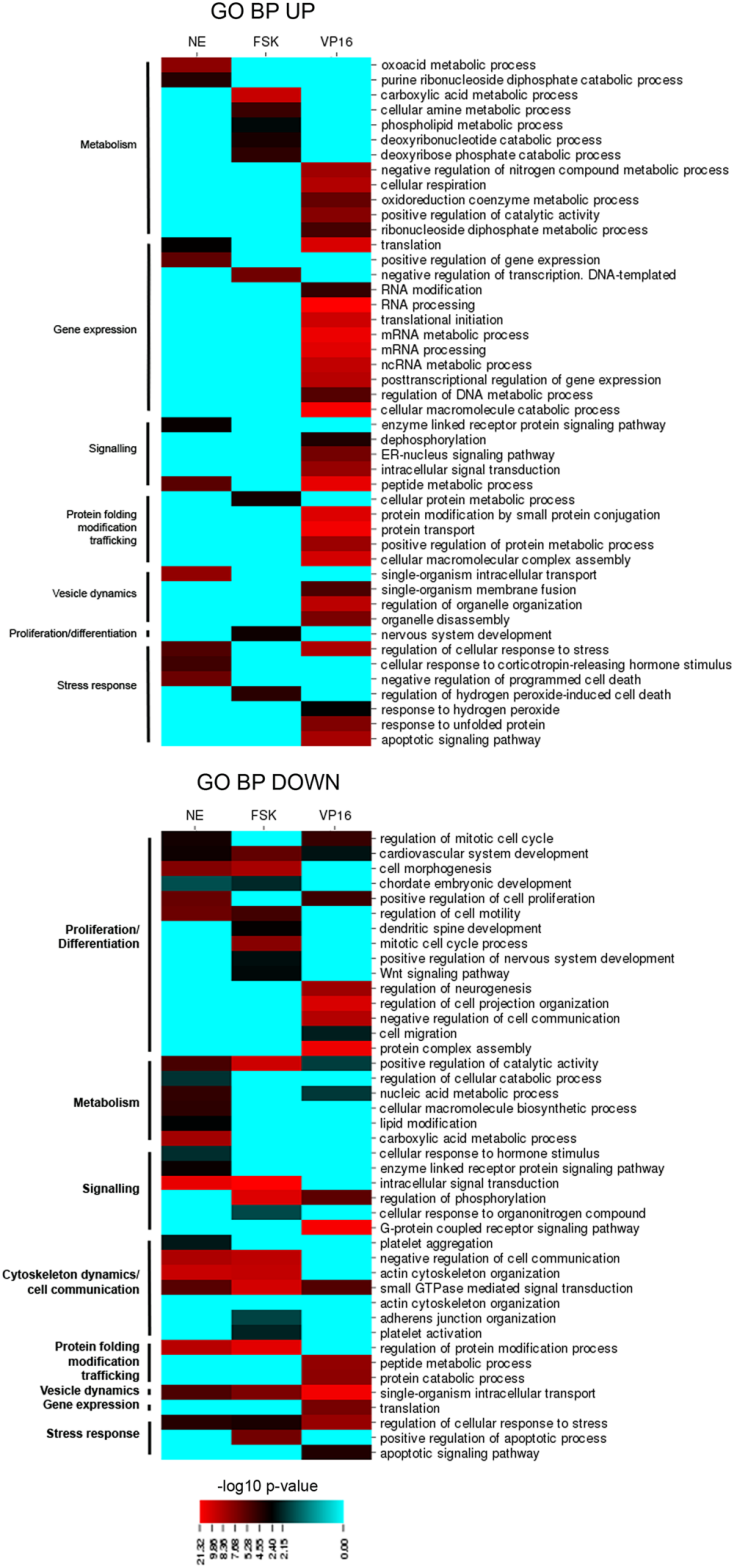
Functional enrichment analysis with GO Biological Pathway. Heatmaps show -log_10_ *p-*values for each enriched GO Biological Pathway (BP) term (described in the right) in the pair-wise comparisons NE-CT; FSK-CT; VP16-Null. GOs were manually grouped into larger categories (left) for better understanding of functional changes among conditions. *p-*values correspond to an enrichment/depletion two-sided hypergeometric statistical test, corrected for multiple comparisons by Bonferroni post-hoc.

**Figure 5:**
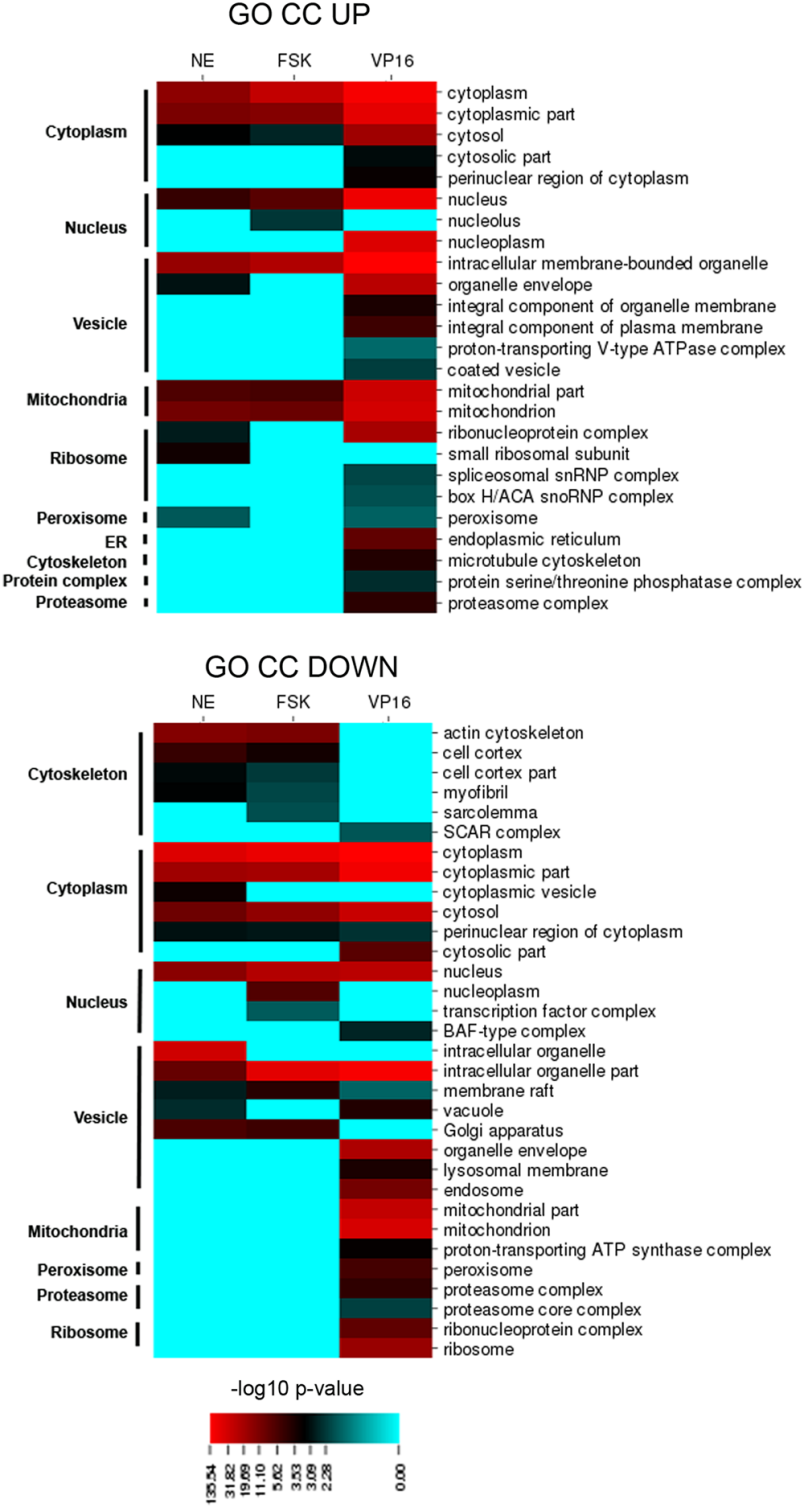
Functional enrichment analysis with GO Cellular Compartment. Heatmaps show -log_10_ *p-*values for each enriched GO Cellular Compartment (CC) term (described in the right) in the pair-wise comparisons NE-CT; FSK-CT; VP16-Null. GOs were manually grouped into larger categories (left) for better understanding of functional changes among conditions. *p-*values correspond to an enrichment/depletion two-sided hypergeometric statistical test, corrected for multiple comparisons by Bonferroni post-hoc.

The transcriptional programs coordinated by ast-CREB were characterized with a GO-based functional enrichment analysis considering categories in Cellular Compartment (CC) and Biological Process (BP) to obtain complementary views. GO was preferred over pathway analysis as with KEGG because we estimate that GO included 85% of our DGEs while KEGG only 40%, leaving a great deal of DEGs uncharacterized. Categories were ranked by significance (−log10 *P*-value) after redundancy was eliminated with ReviGO, and the final categories were manually collapsed in larger themes (Figs 4 and 5). The higher the value, the better represented the category. The complete list of GOs and associated p-values, as well as pathway analysis with KEGG—which largely mirrors the results of GO-BP for V16-CREB, are shown in Supplementary data S3.

Ast-CREB has a profound impact on several functions in astrocytes. Leaving ‘Cytosol’ out because it is too generic, the functional signature comprises seven categories:

1. ‘Gene expression’: This includes ‘Gene expression’ in BP and ‘Nucleus’ in CC, and represents transcription, mRNA processing and translation. Of note, transcription factors are also annotated in categories like ‘RNA processing’. According to significance values, ‘Gene expression’/’Nucleus’ categories are more predominant in the UP section (‘Gene expression’ indeed does not appear in BP-DOWN). The presence of GO categories in ‘Nucleus’ in CC-DOWN in FSK and NE suggests that the transient nature of the latter stimuli may launch a negative feedback of gene expression, absent in the sustained stimulation by VP16-CREB.
2. **‘**Mitochondrial functions’: ‘Mitochondria’ is a clear hit in GO-CC. The categories are more represented in UP compared to DOWN and include nuclear-encoded genes related to mitochondrial functions such as oxidative phosphorylation, tricarboxylic acid cycle (TCA), fatty-acid oxidation, redox homeostasis, replication, transcription, translation, apoptosis regulation, fusion/fission, chaperones, nucleotide homeostasis, and amino acid metabolism, the latter being related to energy metabolism since amino acid catabolism provides intermediates for TCA.
3. ‘Vesicle dynamics’: A highly enriched category is ‘intracellular bounded organelle’, which includes other highly enriched categories like ‘Nucleus’, ‘Mitochondria’, ‘Ribosome’, as well as the endosome system and genes related to SNARE complexes.
4. ‘Stress response’: Pathways include responses against oxidative stress, apoptosis and unfolded protein response, the latter perhaps related to ‘Proteasome’, also present in GO-CC. The significances are generally higher in UP than in DOWN, suggesting that the net tendency is up-regulation of these pathways.
5. ‘Metabolism: This is highly heterogeneous and includes functions like lipid metabolism and nucleic acid metabolic processes (also represented in ‘Gene expression’) and respiration (also represented in ‘Mitochondria’).
6. ‘Signaling’: The most heterogeneous one, featuring different subcategories per group.
7. ‘Proliferation/differentiation/cytoskeleton’: Categories are clearly DOWN.

In summary, ast-CREB triggers intense activity of the transcriptional and translational machinery, associated with general activation of mitochondria functions, vesicle trafficking, modulation of signaling pathways, protection against stress and arrested proliferation. Among the most prominent functions in FSK/NE are amino acid metabolism (‘oxoacid metabolic process’, ‘carboxylic acid metabolic process’) and cytoskeleton dynamics (‘actin cytoskeleton’), while the most relevant functions in VP16-CREB are related to mRNA processing (‘RNA processing’) and proteins (‘translation’, ‘protein transport’, ‘proteosome’).

### Ast-CREB and neu-CREB regulate distinct programs

In parallel we set out to identify *direct* ast-CREB targets upstream from global functional changes. The premises were: (i) gene targets should be concentrated in the UP sections of the post-Q1-TRAP transcriptomes as predicted by the PSCAN analysis (Fig. 2), (ii) harbor CRE-sites in their promoters and (iii) be up-regulated by FSK, NE and VP16-CREB thus representing the *core* targets of ast-CREB independently of specific regulatory contexts.

The transcriptomes of FSK *vs* control, NE *vs* control and VP16-CREB *vs* Null shared 413 genes of which 125 were up-regulated in the three conditions; 71 are listed in the Salk Institute database as CREB-dependent genes^19^. We ranked the genes according to their average fold-change in the FSK, NE and VP16-CREB transcriptomes and listed 13 categories (Supplementary data S4). Most of the categories largely agree with the ones unbiasedly identified in the functional enrichment analyses, namely ‘Gene expression’, ‘Mitochondria’ and ‘Signalling’, ‘Metabolism’, ‘Vesicles’, ‘Redox homeostasis/Antioxidant response’, which is allied to ‘Stress response’, and ‘Protein folding, modification and trafficking’. New categories with more than 4 genes that were not detected or not at the top in the previous screen are ‘Plasticity’ and ‘Hormones’.

At least half of the genes in ‘Gene expression’ are transcription factors. Other nuclear genes are related to ‘RNA export’, ‘DNA replication’ and ‘Translation’. ‘Mitochondria’ comprises genes regulating trycarboxylic acid chain, electron chain, fatty-acid oxidation or mitochondrial translation. *Ndufa5*, a highly conserved NADH: ubiquinone of complex I in the electron chain is the top hit. ‘Signalling’ includes the GTPase *Gem* (top hit). In ‘Metabolism’, the most predominant function is lipid metabolism, as represented by *Dgat*, a key protein in the synthesis of triglycerides. ‘Vesicles’ include genes related to SNARE-dependent exocytosis (*Vamp2*, *Gabarapl2*) or to general endosome trafficking (*Golph3*, *Rhobtb3*, *Reps1*). Finally, ‘Redox homeostasis/Anti-oxidant response’ includes *Hagh*, a thiolesterase responsible for the hydrolysis of S-lactoyl-glutathione to reduced glutathione and D-lactate, protective oxido-reductases like *Cb1*, *Cb3* and *Grhp*, which detoxifies advanced glycation end products and, finally, *Gpx4*, a glutathione peroxidase with a preference for reducing lipid hydroperoxides. ‘Plasticity’ encompasses genes that have been shown to control cell-fate specification and adaptive changes in neurons, and/or to be up-regulated in injury. Since the genes are in the top quartile of the TRAP astrocyte list, and hence highly expressed in adult brain, they may play a role in restorative/repair phenomena including astrocyte proliferation, astrocyte de-differentiation, neuroprotection and circuit remodeling. The category includes genes that protect the blood-brain barrier (*Rdh10*) or promote axon growth (*Ninj1*)^21, 22^. It also includes genes like *Nkd1* and *Galnt119*, respectively involved in Wtn and Notch signalling, pathways also involved in brain injury^23, 24^, and *Rgs2*, recently shown to be involved in signaling and synaptic plasticity in neurons^25^. ‘Hormones’ is represented by *Dio2*, which encodes for a protein highly abundant in adult astrocytes that activates the thyroid hormone and hence may exert a wide set of actions in the brain^26^; other genes are related to steroid metabolism (*Dexi*, *Hsd17B3*). The top gene in ‘Protein trafficking/chaperones’ is the heat shock protein70, *Hspa2*.

Finally, we manually curated the candidate lists in the FSK, NE and VP16-CREB groups in the search for differentially expressed genes associated with well-recognized astrocytic functions such as glutamate/glutamine cycle, neurotransmitter recycling, glutathione metabolism, potassium clearance, maintenance of electrochemical gradients by Na^+^/K^+^ ATPases, ion transport, ApoE metabolism, exocytosis *via* SNAREs, production of growth factors and production of synapse-inducing proteins (Supplementary data S5). The only genes differentially expressed in the three groups, that is, core genes, were genes encoding for SNARE proteins (*Vamp2*, *Vamp5*, *Snap-25*) and *Gpx4*. In summary, of all the canonical astrocyte functions the ones that may be regulated by ast-CREB functions are ‘Vesicle dynamics’ and ‘Redox homeostasis’.

**Figure 6:**
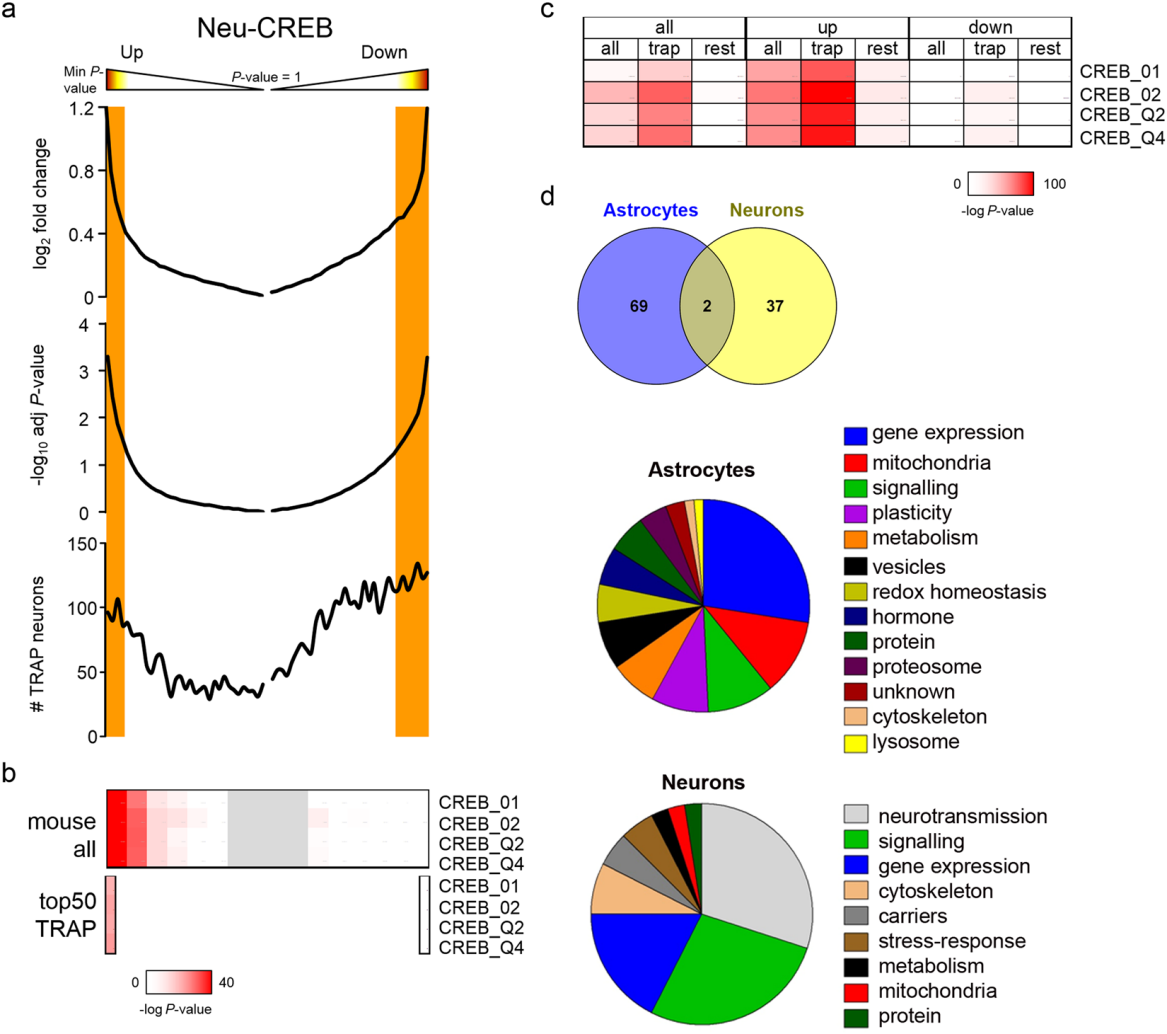
Molecular signatures of CREB in neurons and astrocytes. (**a**) Histograms showing the distribution of genes according to fold change, adjusted p-values, and direction of change (UP or DOWN) in neurons over-expressing VP16-CREB. The whole transcriptome was ranked by the t statistics from more to less significance of change in UP and DOWN sections. Q1-TRAP genes were also mapped below all the transcriptomes, as in Fig. 2. Similar results were observed in the FSK group (not shown). (**b**) PSCAN analysis of CRE-enrichment probability in neurons over-expressing VP16-CREB, using the same procedure as in Fig. 2. CRE-containing genes are enriched in the UP portion, indicating that CREB is mostly a transcriptional activator in neurons. Only the top50 in either UP or DOWN portions were scanned for CRE sites, producing the same bias towards up-regulation. Similar results were observed in the FSK group. (**c**) The same PSCAN analysis without considering bins but entire sets of genes: ‘all’, all the genes; ‘up’ and ‘down’, all the up-regulated and down-regulated genes independently of the significance of change; ‘TRAP’, only the Q1-TRAP list; ‘rest’, the remaining genes. The up-regulated Q1-TRAP genes were enriched in CRE sites. (**d**) Core up-regulated astrocyte genes filtered through the Q1-TRAP list and the CREB-target-gene database (Salk institute) were compared with neuronal databases obtained from neurons treated with FSK or VP16-CREB^10^ processed in like manner. Venn diagrams showed almost no overlapping between both cell types. Categories were manually inferred from gene function according to the literature. The full lists are in Supplementary data S4. Chart slices correspond to the percentage of genes in each category.

We next compared the transcriptional programs of ast-CREB and neu-CREB. To produce comparable materials we processed neuronal lists in the same manner as we did with astrocytes. Thus, the differentially expressed genes of neuronal cultures infected with lentiviruses that transduce VP16-CREB or exposed to FSK^10^ were filtered using the first quartile of the TRAP list to select genes with the highest probability of being relevant to adult function. Figure 6a–c shows the gene distribution in neurons over-expressing VP16-CREB according to expression and statistical significance thereof. Unlike astrocytes, there is some predominance of down-regulated genes that is maintained after the Q1-TRAP filtration (Fig. 6a). However, as with astrocytes, PSCAN revealed enrichment of CRE-sites in the genes derived from the Q1-TRAP list, and more so in the UP group of the differentially expressed. The lower the p-value the greater the density of CRE-sites (Fig. 6c,d).

To generate the core molecular signature of neu-CREB, we identified CREB-target genes in the UP genes common to FSK and VP16-CREB, using the repository of the Salk Institute. There were 93 genes common to FSK and VP16-CREB. Of the 93 genes, 60 were up-regulated in both VP16-CREB and FSK lists, of which 39 appeared in the Salk Institute databases. As expected, core genes regulated by neu-CREB were the ones traditionally associated with CREB in the brain: *Fos*, nuclear receptors (*Nr4a1*, *Nr4a2*), neuropeptides (*Tac1*, *Npy*, *Sst*) and dual specificity phosphatase (*Dusp1*) (Supplementary data S4). The functional categories over-represented in the molecular core of neu-CREB were ‘Neurotransmission’ (12 genes related to well-known neurotransmitters), ‘Signalling’ (11 genes related to kinases/phosphatases) and ‘Gene expression’ (6 out of 7 genes encoding for transcription factors), the top ranking genes being *Tac1*, *Dusp1* and *Nr4a1*. These categories account for 75% of the molecular signature of neu-CREB. Less represented categories are ‘Cytoskeleton’ (*Clstn3*), and ‘Carriers’, with the glucose carrier *Scl2a3*.

Ast-CREB and neu-CREB core signatures thus bear little resemblance. The only common genes are *Impact*, which controls translation, and *Emerin*, a nuclear gene related to actin. Shared categories like ‘Gene expression’ and ‘Signalling’ are represented by different genes.

## Discussion

The purpose of this study was to identify molecular and functional targets of ast-CREB in order to facilitate the study of astrocyte-based adaptive functions of CREB in the brain. A most important finding is that the core signature of CREB differs in astrocytes and neurons. While 75% of the gene signature of neu-CREB encodes for genes related to neurotransmission, signalling and transcription, 75% of the gene signature of ast-CREB controls transcription, mitochondrial functions, lipid metabolism, signalling through MAPK, SNARE function and protective responses encompassing redox protection and repair ‘plasticity’ genes. This difference notwithstanding, the enrichment of CRE-sites in the genes most highly expressed in mature cells—both in neurons and astrocytes—supports that CREB plays as important a function in the adult brain.

It is worth noting that the stringent criteria used to generate signatures (i.e., that genes be differentially expressed by all stimuli and present in the Q1-TRAP list) may leave *bona fide* CREB target genes out. In other words, the existence of core genes does not preclude the context-specific activation of CREB-target genes outside the core. These would include genes activated by cyclic AMP-dependent and independent pathways, here represented by the FSK and NE transcriptomes, which, unexpectedly, were highly similar, suggesting a common downstream step in the signalling pathways, or genes activated by yet unknown physiological pathways, as represented in the VP16-CREB transcriptome, which reveals the ast-CREB ‘regulon’. That said, what the signatures represent are the genes with the greatest probability of being regulated by CREB in astrocytes or neurons, this being a necessary distinction when exploring the role of CREB in whole brains.

An important implication of the results is that they reveal novel ways by which CREB may modulate adaptive processes in the brain such as memory *via* astrocytes. A cornerstone of neuroscience is that learning and memory are the result of the induction of specific genes in neurons in response to experience. CREB is the prototypical memory-related gene, and fulfils the task in large part because it activates other transcription factors such as *Fos*, *Nr4a1*, *Nr4a2* and *Jun*, thereby triggering a broad downstream program which, through structural and functional changes in neurons, ultimately modifies the activity of neuronal circuits^10, 12, 27^. The high representation of transcription factors in the ast-CREB-dependent transcriptomes suggests that ast-CREB is also a hub of activity-driven genetic programs, although through different transcription factors. However, although a wealth of data demonstrates that astrocytes regulate neurotransmission^28^, it is barely recognized that adaptive changes driven by gene expression may happen in astrocytes themselves. Astrocytes have been studied solely in the context of the short-term regulation of synaptic events^29^. Indeed, the available GO classification and pathway enrichment repositories like KEGG mirror the current neuron-centric conceptualization of the brain, and hence lack categories related to astrocyte-specific functions and excitability as shown by calcium responses^30^. This means that the existing databases are obsolete. Their update will require that astrocytic molecules and pathways controlling circuit long-term plasticity be sufficiently conceptualized and characterized to give rise to novel categories. For now, we posit three mechanisms whereby ast-CREB may control adaptive, long-term plasticity in the brain.

One is modulation of gliotransmission. Astrocytes are secretory cells: they respond to neurotransmitters by releasing so-called gliotransmitters (e.g., glutamate, ATP, D-serine) *via* vesicular transporters and Vamp proteins^31^. Vesicle-mediated dynamics and *Vamp2* are hits in functional and molecular signatures of ast-CREB, suggesting that the transcription factor may tune the communication between neurons and astrocytes.

A second mechanism is the modulation of aerobic metabolism. The idea that CREB controls astrocyte energy metabolism stems from the discovery that many ast-CREB hits are genes encoding for mitochondrial proteins. The relationship between CREB and mitochondria is not new. CREB regulates the expression of genes encoding for mitochondrial calcium channels^32^. Also, CREB-regulated transcriptional co-activator (CRTC1) promotes mitochondrial biogenesis via cofactor PGC-1alpha in muscle cells^33^, and down-regulates systemic mitochondrial metabolism via a catecholamine signal originating in neurons^34^. The unifying theme of these studies is that CREB mediates adaptive metabolic changes. Here we posit that, in the brain, adaptive metabolic changes mediated by an ast-CREB/mitochondria axis influence memory formation. This is in line with the emerging notion that mitochondria regulate synaptic plasticity by locally controlling energy status and calcium content^35, 36^. Of note, hits of the array are *Dgat*, related to triglyceride synthesis, which has been recently described as an essential requirement for fatty-acid oxidation in the mitochondria^37^, and *Gpx4*, specialized in reducing lipid-derived reactive oxygen species. All in all, the data suggest that ast-CREB has an impact on astrocyte aerobic metabolism by enhancing mitochondrial fatty-acid oxidation while protecting cells from lipid-derived oxidative stress. Enhanced mitochondrial activation may, in turn, modify astrocyte excitability and hence circuit activity.

A third mechanism is circuit remodeling through genes involved in Wtn and Notch signalling (*Nkd1*, *Galnt119*)^23, 24^, or shown to regulate synaptic plasticity in neurons (*Rgs2*)^25^, raising the possibility that they control astrocyte plasticity as well.

Still, a limitation of the study is that the analyses were performed with materials from cultured neonatal astrocyte and neurons. Although we did find that DEGs from NE/FSK/VP16-CREB astrocytes were enriched in mice with targeted activation of CREB dependent transcription, supporting that the *in vitro* signature is relevant *in vivo*, the possibility remains that neonatal programs, or programs related to maturation, may be present in our DEGs. Along these lines, sustained activation of cyclic AMP-dependent pathways, which may be comparable to our FSK group, has been recently reported to promote maturation of astrocytes^38^, as evidenced by the enrichment of DEGs of astrocytes treated with 8Br-cAMP, an analog of cyclic AMP, in the transcriptome of mature astrocytes^38, 39^. However, over 70% of DEGs in ref. 38 do not overlap with the transcriptome of mature cells^39^, suggesting development-independent actions of cyclic AMP-dependent signaling in astrocytes. Moreover, another recent study reports cyclic AMP/CREB-dependent glutamatergic regulation of mature astrocytes^40^. All in all, the emerging evidence emphasizes the importance of model, and hence of context, in the CREB-dependent regulation of adaptive changes in astrocytes.

In conclusion, we identified here a subset of genes highly expressed in adult astrocytes, differentially regulated upon CREB activation, and harboring CRE-sites at their promoters, indicating that they are direct targets of this transcription factor in astrocytes. Furthermore, we demonstrate that the core signatures of ast-CREB and neu-CREB target genes are different, suggesting novel ways by which CREB regulates brain adaptive functions. Finally, the enrichment of CRE-sites in genes highly expressed in adult astrocytes and neurons supports the relevance of CREB in the regulation of astrocyte-neuron circuits. Future research may identify specific CREB-dependent programs for different physiological contexts.

## Methods

### Cell culture and treatments

Experiments have been carried out in accordance to the guidelines of the European Union Laws for the protection of experimental animals. Experimental protocols have been approved by the Animal Welfare Committee of the Autonomous University of Barcelona and the Generalitat de Catalunya. Cortical astrocyte cultures were prepared from 1-day old Sprague-Dawley rats. Briefly, rats were decapitated and cortices were rapidly removed and separated from brain meninges. The tissue was minced and incubated for 10 min at 37 °C in Ca^2+^-free Krebs-Ringer buffer containing 0.025% trypsin. The cells were then mechanically triturated through a glass pipette and filtered through a 40-µm nylon mesh in the presence of 0.52 mg/ml soybean trypsin inhibitor and 170 IU/ml DNAse. After centrifugation (500 g), the cells were stained with Trypan Blue exclusion, counted in a Neubauer chamber, and then resuspended (3 × 10^5 ^cells/ml) in 90% DMEM, 10% FBS, 20 U/ml penicillin, and 20 µg/ml streptomycin. The cells were seeded in appropriate plates (35 mm dishes for RNA and protein extraction, or in 24-well plates for luciferase assays and immunocytochemistry), and maintained in a humidified atmosphere of 95% air-5% CO_2._ The culture medium was changed two hours after seeding and at the 7th day *in vitro*. Cells were used when confluent, around day 10. Characterization of cellular contents was carried out by immunocytochemistry for astrocyte, microglia, neuron and fibroblast markers (see below).

Treatments with 1 µM FSK and 10 µM NE were performed for 6 hours in confluent cultures by directly adding the agents to the culture medium. The over-expression of VP16-CREB was carried out by infection with the adenoviruses Ad2/5-CMV-VP16-CREB or Ad2/5-CMW (Null) in pre-confluent cultures using DMEM with 1% FBS without antibiotics. The SK-based plasmids were obtained from A. Barco’s laboratory, and the viruses were generated in the Core Service of Virus Production at the Universitat Autònoma de Barcelona. The viruses were tested at a range of multiplicity of infections (MOI) within 5–30. The medium was changed after 3 hours and the cells were used 18 hours post-infection.

### Immunocytochemistry

For immunocytochemistry the cells were fixed in 4% paraformaldehyde for 15 minutes and, after several washes with cold PBS, blocked with 5% NGS for 30 minutes and incubated overnight in a humidified chamber with the primary antibodies against VP16 (1:150, Santa Cruz), GFAP (Dako, 1:2000), NeuN (1:1000, Merck Millipore), Iba1 (1:1000, wAKO) and Vimentin (1:500, Abcam). The next day the cells were incubated with fluorescence-labeled secondary antibodies (Life technologies, 1:1000), counterstained with the nuclear marker DAPI (1:20000, Invitrogen) and mounted with fluoromount-G (Southern biotech). The labeling was visualized with a epifluorescence microscope (Nikon Eclipse 90i) and pictures taken with a NIKON DXM1200F (Sofware ACT-1) at a resolution of 3840 × 3072 pixels and saved in TIF. Pictures were combined using Adobe Photoshop version 8.0.

### Western blot

The cells were lysed in cold RIPA buffer (50 mM Tris-HCl, pH 7.4, 150 mM NaCl, 2 mM EDTA, 0.5% Triton X-100, 1% NP-40, 0.1% SDS, 1 mM Na_3_VO4, 50 mM NaF, and 1 mM PMSF) supplemented with protease and phosphatase inhibitors (Roche). Twenty-five µg of protein was run in 10% SDS-PAGE. After electrophoresis, the proteins were transferred to polyvinyl difluoride membranes, blocked with 5% milk in Tris-buffered saline (TBS) containing 0.1% Tween-20 for 1 h, and incubated overnight at 4 °C with VP16 (Santa Cruz Biotech, 1:150) or actin-β antibodies (Sigma, 1:30000). The next day the membranes were incubated with anti-IgG-horseradish peroxidase-labeled secondary antibodies (Pierce, 1/10,000) for 1 h at room temperature and detected with an enhanced chemiluminescence detection kit (ECL, Amersham Life Science). Films were revealed with Fujifoto FPM-100A, and scanned in HPScanjet G3010.

### Luciferase assays

Pre-confluent astrocyte cultures were transfected with luciferase reporters using FuGene6 (Roche) following manufacturer’s instructions. Briefly, for each well in a 24-well plate, 1 µg of pCRE-luciferase and 0.5 µg of pTK-renilla were mixed with 3 µL of FuGene6 in 250 µL of DMEM 10% FBS without antibiotics. The mixture was incubated for 15 minutes at room temperature and added to the cells. The medium was changed after 2 hours and the cells were subjected to appropriate treatment and monitored for luciferase expression 48 hours after transfection.

### qPCR

The RNA from treated cultures was isolated using the RNAeasy spin kit (Capsumlab) according to the manufacturer’s instructions. Genomic DNA contamination was prevented by treating samples with DNAse1 (Invitrogen). The RNA concentration was determined with the NanoDrop 1000 spectrophotometer (Thermo Scientific) and quality was tested using the Agilent 2100 Bioanalyzer (Agilent Technologies). Only samples with RIN >8 were used. One µg of RNA was reverse transcribed using *Superscript II* reverse transcriptase (Invitrogen) in a reaction of 25 µL. Samples at a 1:100 dilution were amplified in an Applied Biosystems 7500 Fast system using the Power Sybr Green PCR master mix (Life technologies). Data analysis was performed with the comparative Cq method (Pfaffl 2001) using the average value of PCR efficiencies obtained with LinRegPCR software and normalized to *Gapdh*.

### DNA microarray

DNA arrays were carried out by Bioarray® (Alicante, Spain) using SurePrint G3 Rat Gene Expression 8 × 60 K (Agilent, Santa Clara, California, USA). These microarrays include 39,430 Entrez Gene RNAs and 16,251 lincRNAs. The RNA was extracted from cell cultures as described previously. The concentration was determined with the NanoDrop 1000 spectrophotometer (Thermo Scientific), and the RNA quality with Tape Station, using the R6K ScreenTape kit (Agilent). Three samples of each condition (CT, FSK, NE, Null, VP16-CREB) were amplified, labeled for Cy5 or Cy3 using the Low Input Quick Amp Labeling kit (Agilent), and hybridized in the SurePrint G3 Microarray slides according to the manufacturer’s protocol. We performed two independent experiments for the treated and the infected samples, and each replicate was hybridized three times and balanced with respect to the use of Cy3 and Cy5. The microarray slides were scanned using the Agilent microarray scanner (G2565CA), and the resulting images were extracted with Agilent Feature Extraction Software (version 10.7).

### Bioinformatics

Raw data extraction and statistical analyses were performed using Bioconductor packages in the R programming environment. Background correction was performed using the ‘normexp’ method (offset = 10) implemented in the LIMMA package to adjust local median (M) background estimates. Background-corrected intensity data were normalized using the Loess method to remove the bias within each array, and A-value quantile normalization (Aquantile) was used to remove the bias between arrays. The principal component analysis was carried out to ensure the correct separation of the samples. Normalized data were adjusted to a linear model in LIMMA to determine the target genes differentially expressed between groups. P-values were computed with empirical Bayes moderated t-statistics at the level of 0.05 to calculate differential gene expression in the paired comparisons FSK-CT, NE-CT, and VP16-Null. The lists can be found in the Gene Expression Omnibus database (GSE80967). Differentially expressed genes were ranked according to Gene Symbol and t-statistic in absolute value. Mouse orthologous were extracted using the Gene Analyzer Tool from the RGD (Rat Genome Database) website (http://rgd.mcw.edu) to allow for comparisons with the Q1-TRAP and neu-CREB lists, which are from mice.

TRAP lists were retrieved from GSE13379 files. Gene expression was normalized using LIMMA. In the case of the neuronal TRAP lists (below), we averaged the profiles of the several cortical subtypes (Cck+, Pnoc+, cholinergic and neurons from layers 5a, 5b and 6) to minimize their functional and molecular diversity.

Differentially expressed gene lists filtered by the first quartile of the TRAP list (5220 genes) were divided into UP and DOWN depending on the logFC and associated to GO terms (Biological Process and Cellular Compartment) with the software ClueGO v1.4^41^. The analysis was independently conducted in the UP and DOWN lists to gain insight into the predominant direction of change, although it is worth stressing that functional changes normally encompass both up-regulation and down-regulation of genes due to complex homeostatic adjustment of positive and negative feedback loops. The parameters used were enrichment/depletion: two-sided hypergeometric statistical test; correction method: Bonferroni; GO term range levels: 5–10; minimal number of genes for term selection: 3; minimal percentage of genes for term selection: 2%; κ score threshold: 0.5; general term selection method: smallest *p*-value; group method: κ; minimal number of subgroups included in a group: 2; minimal percentage of shared genes between subgroups: 50%. The resulting GO terms were analysed for semantic similarity (cut-off value of 0.4) with ReviGO software^42^ to reduce redundancy, and then manually grouped into categories based on GO ancestors and gene overlapping between terms.

To identify CREB target genes we used PSCAN^18^ to predict CRE-binding sites of the TRANSFAC database in the promoter region (−450/+50) of selected genes, and the database of Salk Institute^19^. Additional software included ‘gplots’ (http://CRAN.R-project.org/package=gplots) and ‘RColorBrewer’ packages (http://CRAN.R-project.org/package=RColorBrewer) to compute heat maps and hierarchical clustering.

GSEA (www.broadinstitute.org/gsea/index.jsp)^20^. Lists of DEG filtered by Q1-TRAP were pre-ranked by fold-change and analyzed for enrichment in gene sets from up-regulated or down-regulated genes in the pairwise comparisons TC-WTC (VP16-CREB lesion vs WT lesion) and WTC-WT (lesion vs control) (GSE68187)^9^. Only enriched sets with a normalized p-value (NOM p-val) < 0.05 were considered significant. Of note, the same platform (Agilent) and procedure was used to identify DEG in the *in vitro* and *in vivo* microarrays.

### Data availabilty

Accession number microarray data. Gene Expression Omnibus GSE80967.

## Electronic supplementary material

Supplementary Information

Supplementary Dataset 1

Supplementary Dataset 2

Supplementary Dataset 3

Supplementary Dataset 4
