# Supplementary material for: CREB Regulates Distinct Adaptive Transcriptional Programs in Astrocytes and Neurons
Source: Sci Rep. 2017 Jul 25;7:6390. doi: 10.1038/s41598-017-06231-x (PMC5526874; doi:10.1038/s41598-017-06231-x)
Supplement: Supplementary file 1 — Supplementary Information [file 41598_2017_6231_MOESM1_ESM.pdf]

CREB Regulates Distinct Adaptive Transcriptional Programs in Astrocytes and Neurons

Luis Pardo*[1], Luis Miguel Valor[3], Abel Eraso-Pichot[1], Angel Barco[2], Arantxa Golbano[1], Giles E. Hardingham[4], Roser Masgrau[1], Elena Galea*[1,5]

1. Institut de Neurociències and Unitat de Bioquímica, Facultat de Medicina, Universitat Autònoma de Barcelona, Bellaterra, 08193 Barcelona, Spain.
2. Instituto de Neurociencias, Universidad Miguel Hernández/Consejo Superior de Investigaciones Científicas, Sant Joan d'Alacant, 03550 Alicante, Spain.
3. Unidad de Investigación, Hospital Universitario Puerta del Mar, Av. Ana de Viya 21, 11009 Cádiz, Spain.
4. Edinburgh Medical School, University of Edinburgh, Edinburgh, EH8 9XD, UK.
5. ICREA, Pg. Lluís Companys 23, 08010 Barcelona, Spain.

Supplementary data

Supplementary figure 1. Full-size western blot of VP16 and actin.

Supplementary excel file 2. Differentially expressed genes upon exposure of astrocytes to FSK, NE or VP16-CREB, shown as genes in Q1-TRAP and rest of genes.

Supplementary excel file 3. Functional analysis with Gene Ontology and KEGG for VP16-CREB.

Supplementary excel file 4. Molecular signatures of ast-CREB and neu-CREB.

Supplementary excel file 5. Differentially expressed genes organized according to canonical functions of astrocytes.

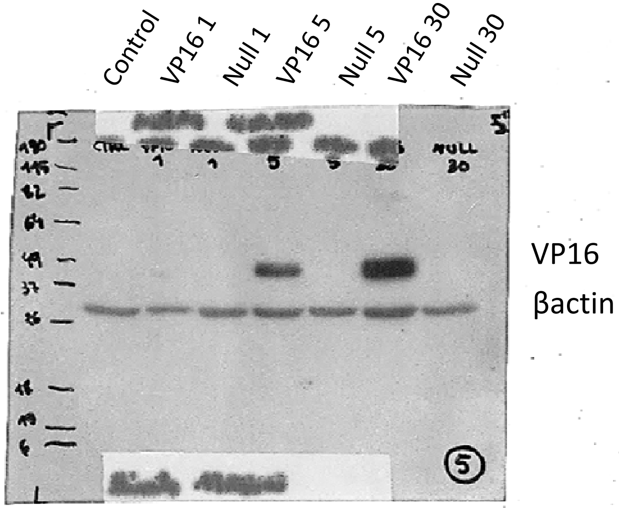
Control
VP16 1
Null 1
VP16 5
Null 5
VP16 30
Null 30
VP16
βactin
